# In vivo staging of regional amyloid progression in healthy middle-aged to older people at risk of Alzheimer’s disease

**DOI:** 10.1186/s13195-021-00918-0

**Published:** 2021-10-21

**Authors:** Fedor Levin, Irina Jelistratova, Tobey J. Betthauser, Ozioma Okonkwo, Sterling C. Johnson, Stefan J. Teipel, Michel J. Grothe

**Affiliations:** 1 German Center for Neurodegenerative Diseases (DZNE), Rostock/Greifswald, Rostock, Germany; 2 Division of Geriatrics and Gerontology, Department of Medicine, University of Wisconsin–Madison School of Medicine and Public Health, Madison, WI USA; 3 Wisconsin Alzheimer’s Disease Research Center, University of Wisconsin–Madison School of Medicine and Public Health, Madison, WI USA; 4 Wisconsin Alzheimer’s Institute, University of Wisconsin–Madison School of Medicine and Public Health, Madison, WI USA; 5 Geriatric Research Education and Clinical Center, William S. Middleton Memorial Veterans Hospital, Madison, WI USA; 6 Department of Psychosomatic Medicine, University of Rostock, Rostock, Germany; 7 Unidad de Trastornos del Movimiento, Servicio de Neurología y Neurofisiología Clínica, Instituto de Biomedicina de Sevilla, Hospital Universitario Virgen del Rocío/CSIC/Universidad de Sevilla, s/n, 41013 Seville, Spain

**Keywords:** Amyloid-β, Amyloid PET, ^11^C-PiB, Amyloid staging

## Abstract

**Background:**

We investigated regional amyloid staging characteristics in ^11^C-PiB-PET data from middle-aged to older participants at elevated risk for AD enrolled in the Wisconsin Registry for Alzheimer’s Prevention.

**Methods:**

We analyzed partial volume effect-corrected ^11^C-PiB-PET distribution volume ratio maps from 220 participants (mean age = 61.4 years, range 46.9–76.8 years). Regional amyloid positivity was established using region-specific thresholds. We used four stages from the frequency-based staging of amyloid positivity to characterize individual amyloid deposition. Longitudinal PET data was used to assess the temporal progression of stages and to evaluate the emergence of regional amyloid positivity in participants who were amyloid-negative at baseline. We also assessed the effect of amyloid stage on longitudinal cognitive trajectories.

**Results:**

The staging model suggested progressive accumulation of amyloid from associative to primary neocortex and gradually involving subcortical regions. Longitudinal PET measurements supported the cross-sectionally estimated amyloid progression. In mixed-effects longitudinal analysis of cognitive follow-up data obtained over an average period of 6.5 years following the baseline PET measurement, amyloid stage II showed a faster decline in executive function, and advanced amyloid stages (III and IV) showed a faster decline across multiple cognitive domains compared to stage 0.

**Conclusions:**

Overall, the ^11^C-PiB-PET-based staging model was generally consistent with previously derived models from ^18^F-labeled amyloid PET scans and a longitudinal course of amyloid accumulation. Differences in longitudinal cognitive decline support the potential clinical utility of in vivo amyloid staging for risk stratification of the preclinical phase of AD even in middle-aged to older individuals at risk for AD.

**Supplementary Information:**

The online version contains supplementary material available at 10.1186/s13195-021-00918-0.

## Introduction

Aggregates of amyloid-β (Aβ) protein are an important early histopathological hallmark of Alzheimer’s disease (AD). Previous research has demonstrated that early amyloid accumulation can be observed in cognitively healthy people long before the onset of dementia [1–4]. Deposition of amyloid in the brain can be measured in vivo using positron emission tomography (PET) with amyloid-sensitive radiotracers such as ^11^C Pittsburgh Compound B (PiB) or 2nd-generation ^18^F-labeled radiotracers. These tracers show high sensitivity and specificity when compared to the neuropathological gold standard [5–8]. However, in contrast to the established neuropathological staging schemes of regionally progressing amyloid pathology [4, 9], clinical PET-based in vivo assessment of amyloid pathology is most commonly limited to a binary classification into positive or negative categories based on the global amyloid PET signal.

In a previous study, we developed a data-driven in vivo staging model of regional amyloid progression which was based on the frequency of regional amyloid positivity in ^18^F-florbetapir PET scans of cognitively unimpaired older adults [10]. The staging model was also validated in an independent cohort of participants with subjective cognitive decline [11]. In both studies, amyloid staging identified early stages of amyloid accumulation that were not detected by the conventional binary classification approach based on global PET signal. In an imaging-to-autopsy correlation study, the staging was also associated with neuropathologically defined phases of amyloid deposition [12]. Moreover, a stage-proportional risk for clinical disease progression could be demonstrated across cohorts [13]. Taken together, these findings indicate the potential usefulness of in vivo amyloid staging for a pathologic stratification of preclinical AD.

However, it is clear that the nuances of the methodological approach, such as radiotracer selection, definition of amyloid positivity cutoffs, choice of reference region, and the use of partial volume effects (PVE) correction, can affect the quantitative amyloid PET imaging results and the regional staging outcomes [8, 14, 15]. Analysis of amyloid-PET scans of young healthy adults who are highly unlikely to exhibit cerebral amyloid deposition demonstrated a considerable variation in regional uptake values [16]. This indicates regionally varying noise levels in amyloid-PET signals and argues for the use of region-specific amyloid positivity cutoffs in the staging model [17]. It was previously reported that global cortical signals of ^11^C-PiB-PET and ^18^F-based amyloid-PET imaging data obtained from the same individuals were highly correlated [18]. However, subtle differences in binding affinity to amyloid and non-specific white matter binding between different tracers have also been demonstrated [19–22], which could potentially affect their ability to detect early diffuse and later neuritic types of Aβ protein aggregates and could thus also result in differing regional patterns of early-stage amyloid pathology. Finally, the characteristics of the studied cohort could likely affect the outcome of the analysis. Particularly, age is expected to influence the ability of amyloid staging to predict cognitive decline.

In the present study, we determined the regional amyloid staging characteristics in dynamically acquired ^11^C-PiB-PET data from middle-aged to older individuals enrolled in the Wisconsin Registry for Alzheimer’s Prevention (WRAP) study who are at increased risk for AD due to a family history of AD dementia. Of note, this cohort was considerably younger than the cohorts investigated previously. We further assessed the longitudinal validity of the cross-sectionally estimated staging model by analyzing individual stage transitions in serial PET scans and additionally analyzed the first sites of longitudinal amyloid accumulation in participants without any evidence of regional amyloid positivity at baseline. Finally, we assessed whether cross-sectionally estimated amyloid stages were predictive of longitudinal cognitive trajectories in this relatively young at-risk cohort.

## Materials and methods

### Participants

We studied 220 participants (mean age = 61.4 years, range 46.9–76.8 years, 151 females) selected from the WRAP cohort based on the availability of at least one ^11^C-PiB-PET scan. Participants underwent the first ^11^C-PiB-PET scan on average 6.4 years after the first neuropsychological assessment. Among participants, 159 (72%) had at least one biological parent diagnosed with AD dementia. A subset of 157 participants had available imaging data from a second ^11^C-PiB-PET scan (average time interval of 2.5 years since the first ^11^C-PiB-PET scan), and 60 had additional imaging data from a third ^11^C-PiB-PET scan (average time interval of 6.1 years since the first scan). Participants were healthy and unimpaired at baseline. Eight participants had a diagnosis of clinical MCI at a WRAP study visit prior to the first ^11^C-PiB-PET scan, and one had a dementia diagnosis shortly after the first ^11^C-PiB-PET scan. We excluded the data from these participants from the longitudinal analysis of cognitive performance. Out of 189 participants with available *APOE*-ε4 data, 34% were *APOE*-ε4 positive.

### Imaging data

Acquisition of ^11^C-PiB-PET and MRI imaging data in the WRAP cohort has been described in detail previously [23]. Briefly, ^11^C-PiB-PET scans were acquired in a 3-D mode with a dynamic 70-min acquisition protocol after an injection of a 15-mCi target dose of ^11^C-PiB bolus. Dynamic acquisition frames consisted of 17 time frames, including 5 × 2 min and 12 × 5 min frames. A filtered back-projection algorithm was used for reconstructing the data. For anatomical reference, a high-resolution T1-weighted MRI scan was acquired using a 3.0-Tesla GE MR750 scanner with an 8 or 32 channel head coil. The 3-D inversion recovery prepared fast spoiled gradient-echo sequence had the following parameters: inversion time (TI) = 450 ms, repetition acquisition matrix = 256 × 256 × 156 mm, field of view (FOV) = 256 mm, and slice thickness = 1.0 mm. The reconstructed time series of ^11^C-PiB-PET data were realigned, corrected for motion, de-noised, and coregistered to the subject’s T1-weighted MRI scan based on co-registration of the time-integrated PET scan utilizing the Statistical Parametric Mapping software (SPM12; www.fil.ion.ucl.ac.uk/spm). Parametric distribution volume ratio (DVR) maps were generated using Logan graphical analysis methods [24, 25] with t* = 35 min and cerebellar gray matter as a reference region of non-displaceable binding.

### Image analysis

The imaging data were further pre-processed for regional staging analysis using previously described procedures [10]. MRI images were segmented into different tissue types and spatially normalized to a customized aging/AD-specific reference template space [26] using the high-dimensional spatial registration algorithm DARTEL [27]. ^11^C-PiB-PET DVR maps were corrected for PVE using the 3-compartment “Müller-Gärtner” method in the subject’s native space [28, 29], and then spatially normalized to the reference template space using transformation parameters from the corresponding MRI. Regional DVR values were then extracted from 52 regions of interest within the reference template defined using the Harvard-Oxford atlas, which included 48 cortical regions, as well as the hippocampus, amygdala, striatum, and thalamus. We also extracted the average global non-PVE-corrected and PVE-corrected ^11^C-PiB-PET DVR signal within a cortical composite mask [30].

We used a two-dimensional Gaussian mixture model (GMM) approach utilizing regional and global mean PVE-corrected DVR values to establish region-specific thresholds for amyloid positivity. Analogous to previous studies using one-dimensional GMM [8, 31, 32], we fit low and high amyloid distributions for each region. The two-dimensional GMM approach is different in that it estimates the distribution of two variables at once so that the contribution of each regional DVR value to the low or high amyloid distribution is estimated in conjunction with the global amyloid signal of each participant. This approach was intended to decrease the susceptibility of the procedure to the potential noisiness of regional signal resulting in more robust and biologically plausible regional estimates. Regional thresholds were defined as 1.65 standard deviations above the mean value of the low Aβ distribution corresponding to the 95th percentile [16, 33].

In analogy to neuropathological staging models and our previous PET-based staging study, we determined a regional amyloid progression model based on the frequency of regional amyloid positivity across individuals as an indicator of progressive temporal involvement [4, 9, 10, 34]. Regional frequencies of amyloid positivity were calculated from the baseline PET data using 10,000 bootstrap resamples, and the obtained range of frequencies was split into four equal parts to obtain a discrete stage model of amyloid progression across four larger anatomical divisions [10, 17].

In sensitivity analyses, we additionally assessed the effect of alternative PET processing methods as well as strategies for estimating regional positivity thresholds. Among PET processing strategies, we assessed the effects of (i) using more commonly obtained standard uptake value ratio (SUVR) images [8, 21] instead of the DVR images obtained from the dynamic PET acquisitions, (ii) using non-PVE-corrected PET data, and (iii) using a constant vs region-specific thresholds. Alternative strategies for estimating regional positivity thresholds included (i) a 1-dimensional GMM approach based on regional values only and (ii) a regional resampling approach in a subsample of the 20 youngest, *APOE*-ε4-negative subjects without familial history of AD (mean age = 59.8 years, 16 females). For both of these methods, the thresholds were analogously estimated as 1.65 standard deviations above the mean value, and regional frequencies of amyloid positivity were calculated using 10,000 bootstrap resamples. The correspondence between the regional amyloid positivity frequencies derived from the different PET processing and cutoff derivation methods was assessed using pair-wise Spearman rank correlations.

Individual amyloid deposition profiles were staged according to the regional hierarchy indicated by the estimated amyloid progression model. For that, each of the four larger anatomical divisions defined by the 4-stage model was considered amyloid-positive if at least half of the included regions displayed a suprathreshold signal [10, 17]. The individual stage was then determined based on the corresponding amyloid-positive anatomical divisions. For example, a classification of stage III requires positivity in anatomical divisions 1, 2, and 3, but not 4. Participants whose regional amyloid positivity profile did not adhere to the expected regional hierarchy (e.g., positivity in anatomical division 2, but not in 1) were classified as non-stageable. For comparison, we dichotomized the ^11^C-PiB-PET scans into standard amyloid-positive/negative categories based on a previously established threshold of 1.08 applied to the global composite DVR value in non-PVE-corrected data [8].

### Longitudinal imaging analysis

The longitudinal validity of the cross-sectionally estimated regional amyloid staging model was assessed in two complementary analyses. First, we assessed individual longitudinal changes in amyloid stages from baseline to the furthest available follow-up PET scan. Among stageable participants at baseline, 155 had a follow-up ^11^C-PiB-PET scan with an average time delay of 4 years (range 1.7–7.7). In a complementary analysis independent from the estimated staging model, we assessed the first longitudinal appearance of regional amyloid positivity in subjects who had no suprathreshold signal in any of the 52 brain regions at baseline (*n* = 64) by recording the regional amyloid positivity occurring at the follow-up ^11^C-PiB-PET scans, on average 3.9 years later (range 1.8–7.6 years).

### Neuropsychological testing

Finally, to examine the potential clinical relevance of the amyloid staging approach, we analyzed the longitudinal cognitive trajectories of participants at different amyloid stages using previously developed domain-specific and global cognitive composite scores [35]. These scores included a delayed recall composite (THEO-DEL-REC), an executive function composite (THEO-EXEC-FN), an immediate learning composite (THEO-IMM-LRN), and a global cognitive composite score—a three test version of the preclinical Alzheimer’s cognitive composite (PACC3).

In order to assess the differences in future cognitive trajectories across in vivo amyloid stages, in the regression analysis, we selected neuropsychological scores obtained at visits taking place at the earliest 3 months before the first ^11^C-PiB-PET measurement and later. Five participants did not have available neuropsychological test scores after that time point and were excluded. The composite scores were only available from WRAP study visit 2 and onwards, because the more extensive cognitive testing required for the composite score calculation was not yet introduced at the first WRAP study visits. As a result, the closest WRAP visit with neuropsychological testing was on average 1.1 years after the first ^11^C-PiB-PET scan. We included cognitive data from a median of 3 WRAP study visits per participant conducted on average at 2.5-year intervals. The mean duration of the total follow-up was 6.5 years from the first ^11^C-PiB-PET scan until the last available cognitive assessment, with a maximum of 8.7 years of follow-up. Longitudinal trajectories of the four cognitive composite scores were analyzed using linear mixed-effects regression models implemented in R 3.6.0 [36]. The effect of the amyloid stage on longitudinal cognitive decline was assessed by the time × amyloid stage interaction, controlled for age at the first analyzed WRAP visit, sex, and years of education.

## Results

### Frequency-based staging model of regional amyloid progression

**Fig. 1:**
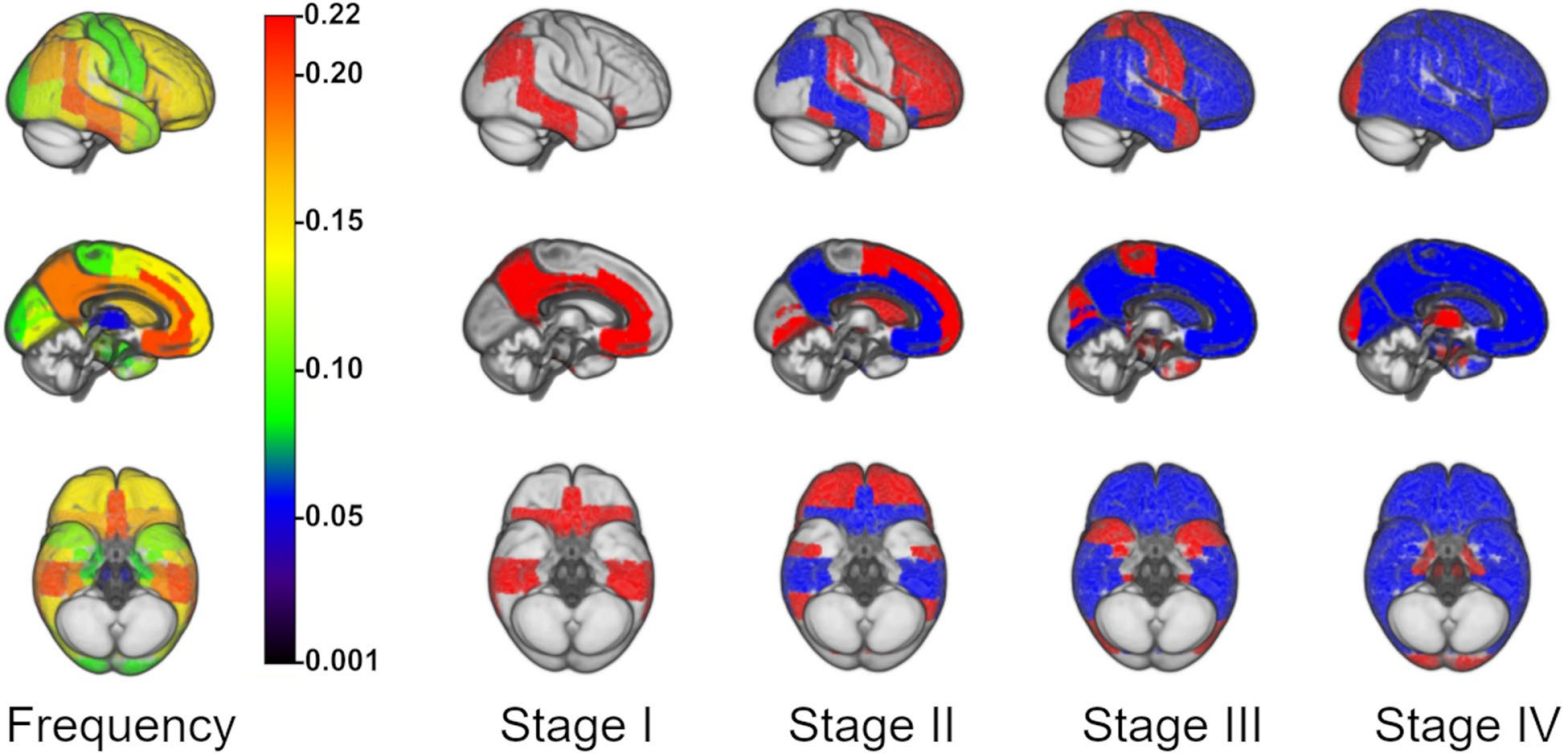
Regional amyloid progression model and the derived staging scheme with 4 stages. Brain renderings on the left illustrate the frequency of regional amyloid positivity (color scale) from black/blue (lowest) to yellow/red (highest), which was used as an indicator of temporal progression. In the resulting staging scheme on the right, incremental stages (I–IV) are defined by an involvement of higher numbered anatomic divisions (in red) in addition to the affected areas of the previous stage (blue)

Overall, 133 out of the 220 participants (60%) demonstrated amyloid positivity in at least one brain region. According to the cross-sectional amyloid progression model based on regional frequencies (Fig. 1), amyloid deposition begins in the anterior and posterior cortical midline structures, the inferior temporal lobe, and lateral temporo-parietal association areas (stage I); stage II involves more extensive parts of the association cortex, particularly the lateral frontal areas, as well as the striatum; stage III is characterized by involvement of primary sensory-motor areas (pre- and postcentral gyrus), as well as of the medial temporal lobe (including hippocampus and amygdala) and temporal pole; and stage IV finally includes the occipital pole, remaining parts of the medial temporal lobe, and the thalamus.

We observed moderate to strong positive correlations between regional amyloid positivity frequencies estimated with three different methods for defining regional amyloid positivity thresholds (see Supplementary table 1 and Supplementary figure 1). In general, the two-dimensional and one-dimensional GMM approaches yielded highly correlated regional progression models (rho = 0.78), whereas larger differences were observed when thresholds were derived from the 20 youngest participants (Spearman rank correlations rho = 0.63 and 0.57, compared to 2D-GMM and 1D-GMM, respectively). Some of the most salient differences corresponded to a relatively earlier involvement (i.e., higher relative amyloid positivity frequencies) of some temporal lobe (Heschl’s gyrus and planum temporale) and subcortical regions (thalamus, hippocampus, and amygdala; see Supplementary figure 1 for brain renderings of the respective amyloid positivity frequencies). We observed a moderate correlation between the current ^11^C-PiB-PET-based model and the previous regional frequency-based model derived from ^18^F-Florbetapir-PET data in older cognitively normal participants from the ADNI cohort [10] (rho = 0.54, *p* < 0.001). However, notable differences between the models were also evident, particularly, with respect to a relatively earlier involvement of medial parietal (stage I) and striatal regions (stage II) in the current ^11^C-PiB-PET-based model.

The results of the individual staging analysis and sample characteristics by stage are presented in Table 1. Thirty-seven participants (17%) were amyloid-positive in any anatomical division, and four of these participants were non-stageable. As expected, higher amyloid stages corresponded to higher mean global DVR values, and 1.6% of stage 0 participants, 36.7% of stage I participants, and all of stage II, stage III, and stage IV participants were classified as amyloid-positive based on the standard global amyloid signal threshold (Table 1). Two of the four non-stageable participants were also classified as amyloid-positive.

**Table 1:** Sample characteristics by regional amyloid stage

	Full sample	Stage 0	Stage I	Stage II	Stage III	Stage IV	Non-stageable
*n*	220	183	8	7	10	8	4
Age, years (*SD*)	61.4 (6.2)	60.7 (6.2)	67.5 (6.0)	64.5 (3.5)	63.7 (2.8)	65.2 (4.8)	64.7 (8.9)
Sex, % female	69%	68%	75%	57%	80%	75%	50%
Education, years (*SD*)	16.4 (2.6)	16.4 (2.7)	16.8 (1.8)	17.6 (2.0)	16.9 (2.6)	15.9 (2.3)	15.3 (2.2)
APOE ε4 (%)	34%	29%	40%	83%	60%	71%	0%
Parental history of AD (%)	73%	71%	43%	86%	90%	100%	75%
Mean global ^11^C-PiB DVR	1.03	0.98	1.08	1.19	1.32	1.46	1.07
Global ^11^C-PiB DVR > 1.08	33 (15%)	3 (1.64%)	3 (37.5%)	7 (100%)	10 (100%)	8 (100%)	2 (50%)
MMSE	29.3 (1.1)	29.3 (1.1)	29.7 (0.5)	29.4 (0.8)	29.4 (0.8)	28.5 (2.7)	29.8 (0.5)
Delayed recall composite score	0.04 (0.85)	0.07 (0.81)	− 0.02 (0.83)	0.23 (0.92)	− 0.27 (0.84)	− 0.59 (1.73)	0.09 (0.96)
Executive function composite score	− 0.06 (0.71)	− 0.01 (0.69)	0.22 (0.25)	− 0.43 (1.30)	− 0.60 (0.56)	− 0.63 (0.54)	− 0.06 (0.52)
Immediate learning composite score	0.04 (0.82)	0.06 (0.80)	0.11 (0.71)	0.21 (0.89)	− 0.27 (0.96)	− 0.27 (1.09)	0.25 (1.11)
PACC3 composite score	− 0.03 (0.78)	0.00 (0.78)	0.29 (0.55)	0.07 (0.69)	− 0.59 (0.79)	− 0.43 (0.93)	− 0.05 (0.86)

Values for age, years of education, MMSE and cognitive composite scores are presented as means with standard deviation in parentheses. Please note that individuals with missing values were excluded from this summary

### Longitudinal analyses of regional amyloid progression

**Fig. 2:**
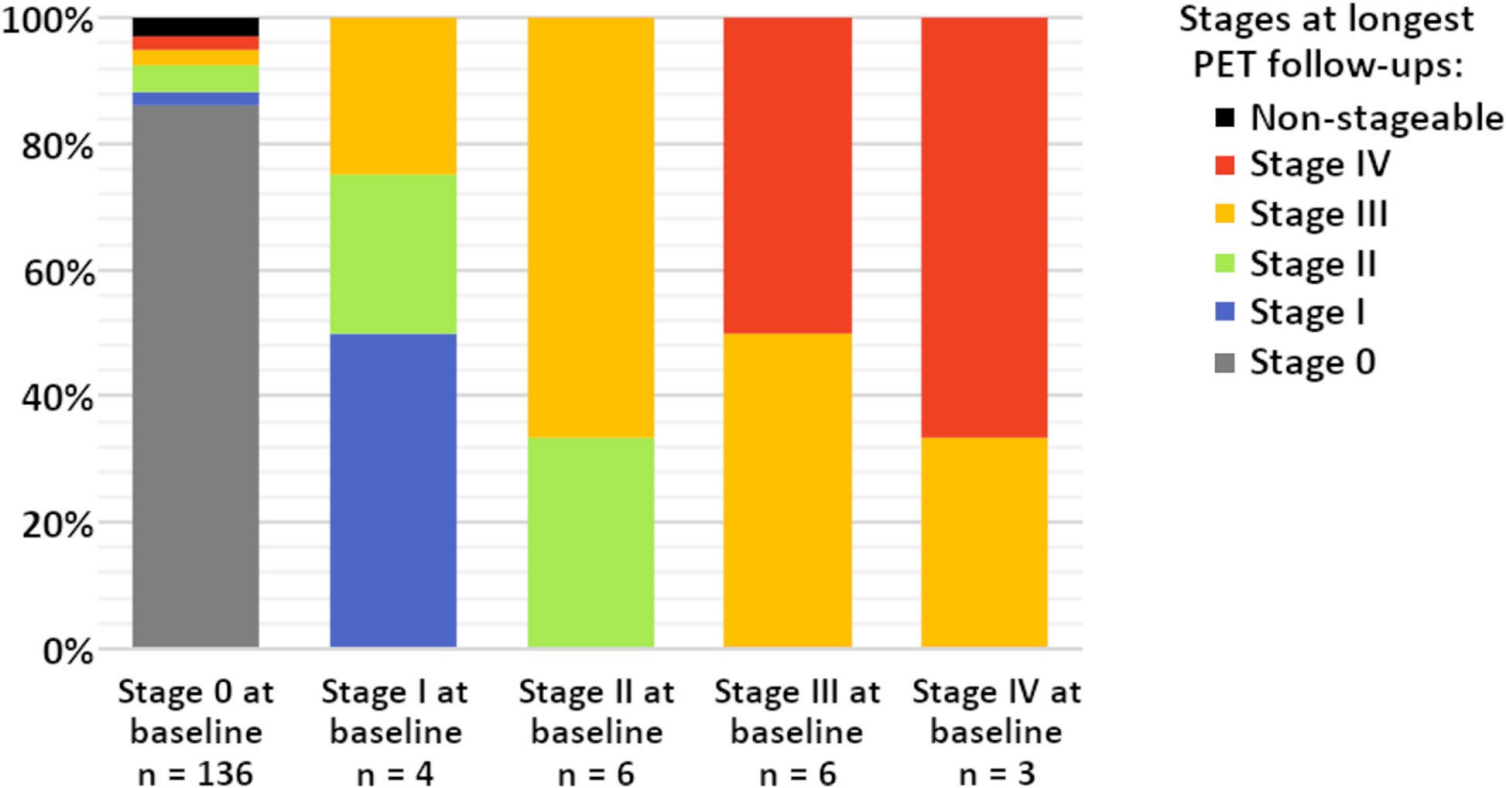
Proportions of in vivo amyloid stages at PET follow-up according to the amyloid stage at baseline. Amyloid stages at follow-up are calculated for the longest available PET follow-up. Participants non-stageable at baseline were excluded

Comparison of the stages determined at baseline and follow-up PET scans suggested that participants in each stage either remained stable at the baseline stage (81.3% of 155 stageable participants with a follow-up scan, see Fig. 2) or showed a model-conform transition to a higher stage (15.5%). One participant demonstrated a regression from stage IV at baseline to stage III at follow-up, and four participants (2.6%) showed a progression pattern that violated the estimated regional hierarchy, i.e., classified as non-stageable at follow-up (all stage 0 at baseline). Characteristics of the subsample of participants who progressed to a higher stage compared to those who did not are presented in Supplementary table 2.

**Fig. 3:**
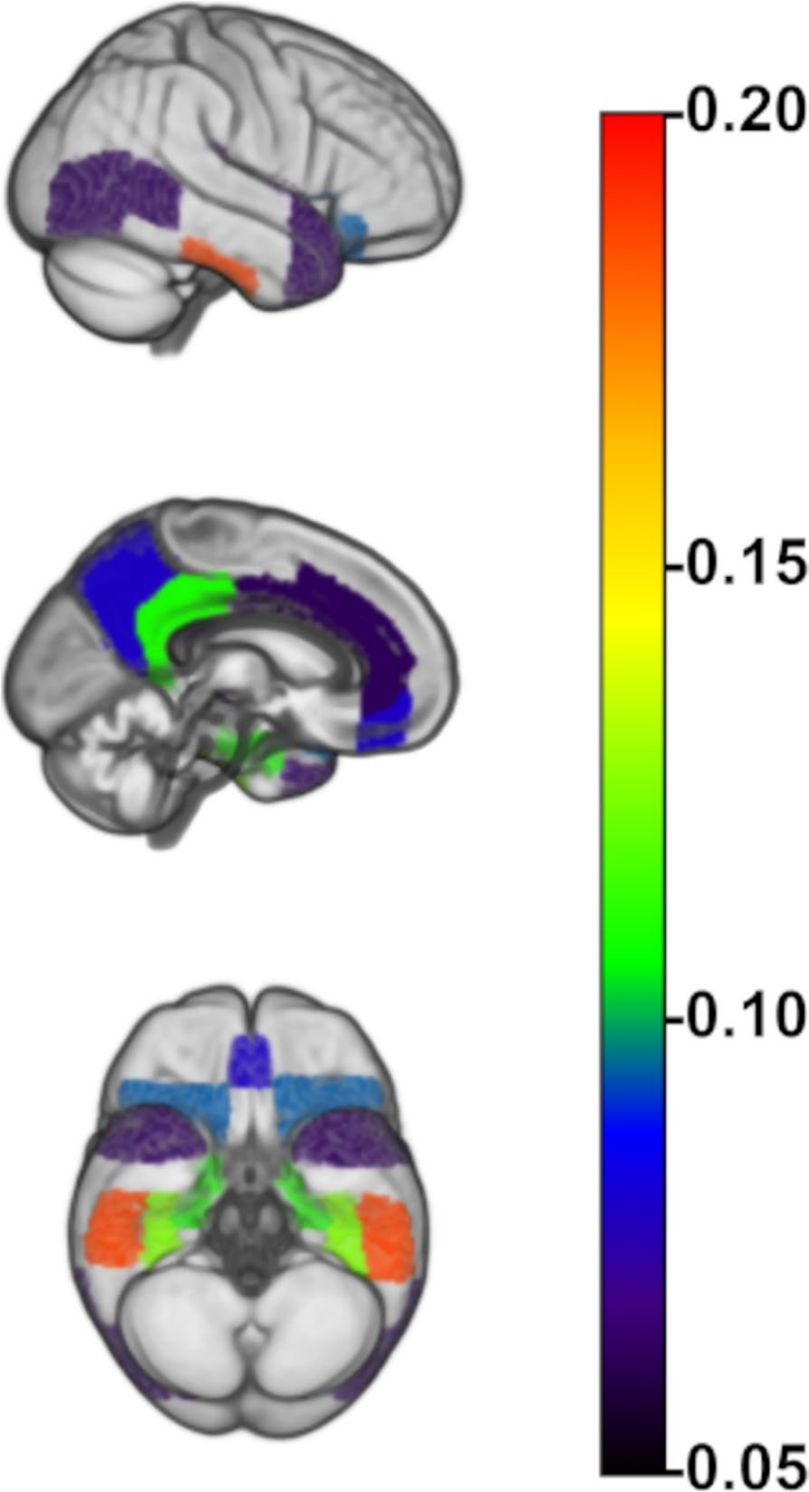
The first longitudinal appearance of regional amyloid positivity. Color scale reflects the probability of a brain region to become amyloid-positive over the longest available PET follow-up in participants who were completely amyloid-negative at baseline. Only regions with probabilities higher than 0.05 are shown

In the longitudinal analysis of participants who had no amyloid-positive regions at baseline and had follow-up scans (*n* = 64), 35 participants (55%) developed amyloid positivity in one or more regions over the follow-up period. Regional emergence of amyloid positivity was mostly localized to the inferior temporal (18.8%) and temporal fusiform gyrus (12.5%), the anterior parahippocampal gyrus (10.9%), and the posterior cingulate cortex (10.9%) but was also observed in the medial frontal and lateral temporal areas in smaller subsets of individuals (see Fig. 3).

### Effect of amyloid stage on cognitive decline

**Fig. 4:**
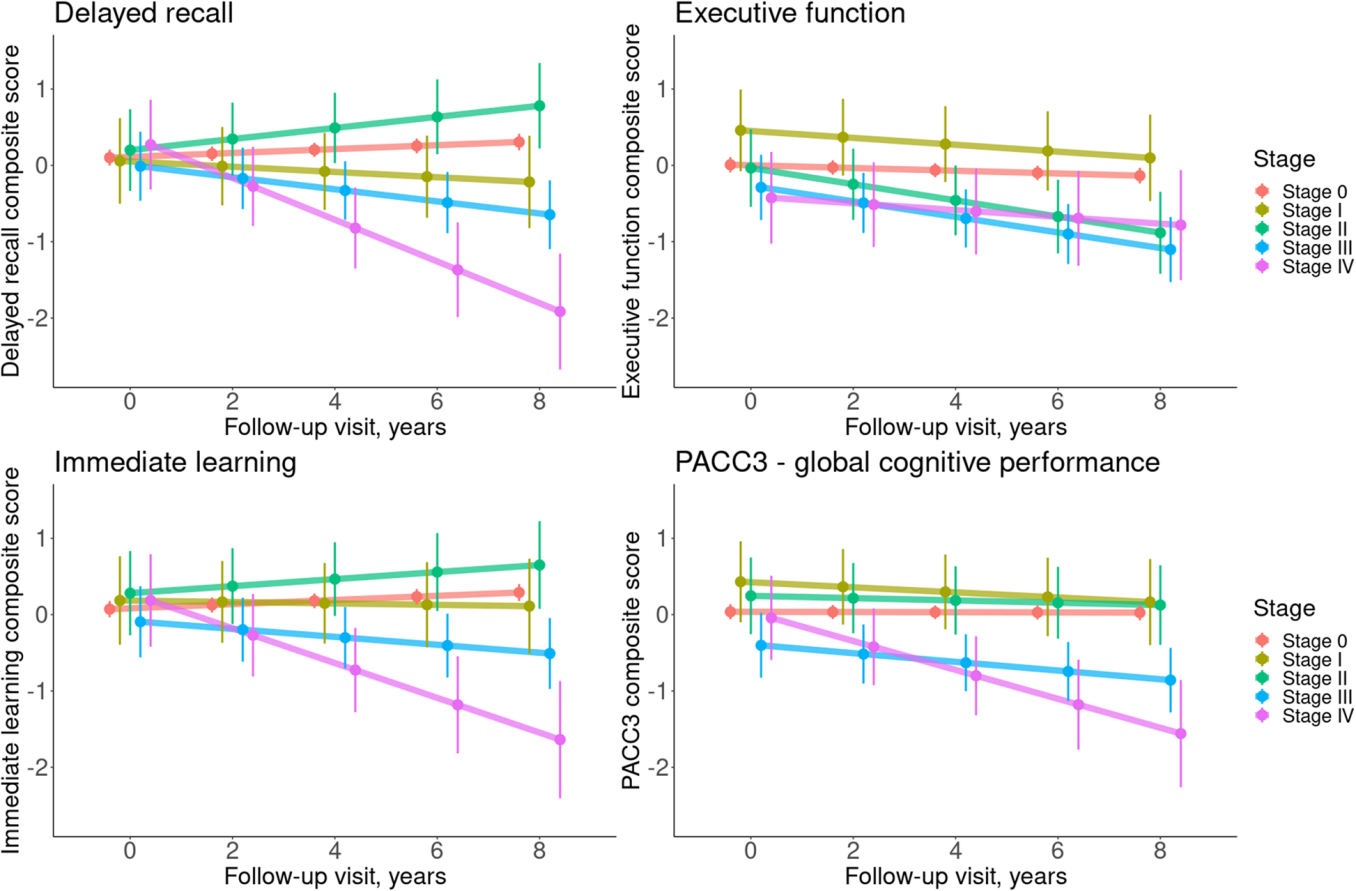
Longitudinal cognitive trajectories of amyloid stages. Plots of composite cognitive scores predicted from the mixed-effects regression models of longitudinal change in composite scores across participants at different in vivo amyloid stages. Please note that actual follow-up intervals differed among participants, and 2-year intervals were used here for demonstration. Error ticks represent 95% confidence intervals

Results from the mixed-effects regression models indicated differential trajectories of cognitive decline depending on the baseline amyloid stage (Fig. 4, Table 2). Compared to stage 0, stages II and III showed a faster decline in executive function, whereas stages III and IV showed a faster decline in immediate learning, delayed recall, and global cognition as measured by the PACC3 score.

**Table 2:** Mixed-effects regression models of longitudinal change in composite cognitive scores across baseline amyloid accumulation stages

	Delayed recall composite score		Executive function composite score		Immediate learning composite score		PACC3 composite score	
	Estimate	*t*-statistic	Estimate	*t*-statistic	Estimate	*t*-statistic	Estimate	*t*-statistic
Intercept	− 0.338	− 0.542	2.829***	4.557	− 0.051	− 0.078	0.567	0.934
Age	− 0.021**	− 2.683	− 0.06***	− 7.765	− 0.029***	− 3.555	− 0.04***	− 5.344
Gender	0.482***	5.05	0.103	1.069	0.565***	5.655	0.528***	5.684
Education	0.054**	3.214	0.039*	2.344	0.056**	3.184	0.063***	3.826
Follow-up time, years	0.026***	3.889	− 0.018**	− 3.294	0.027***	4.218	− 0.001	− 0.256
Stage I	− 0.043	− 0.149	0.451	1.624	0.114	0.378	0.393	1.43
Stage II	0.099	0.356	− 0.043	− 0.164	0.21	0.731	0.209	0.799
Stage III	− 0.113	− 0.479	− 0.297	− 1.326	− 0.166	− 0.68	− 0.44*	− 1.981
Stage IV	0.171	0.56	− 0.433	− 1.397	0.114	0.364	− 0.08	− 0.281
Follow-up time × stage I	− 0.06	− 1.593	− 0.027	− 0.897	− 0.037	− 0.99	− 0.032	− 1.007
Follow-up time × stage II	0.047	1.227	− 0.088**	− 2.712	0.019	0.5	− 0.014	− 0.425
Follow-up time × stage III	− 0.105***	− 3.377	− 0.084***	− 3.348	− 0.079**	− 2.598	− 0.056*	− 2.132
Follow-up time × stage IV	− 0.299***	− 5.514	− 0.027	− 0.596	− 0.255***	− 4.783	− 0.188***	− 4.043

Unstandardized estimates are presented with *t*-statistics. *p < .05, **p < .01, ***p < .001. For interactions between the follow-up time in years and stage, the stage 0 group acts as a reference. Random intercepts for participants are included to account for multiple measurements

## Discussion

In the current study, we established a regional staging model of progressive amyloid accumulation in cross-sectional ^11^C-PiB-PET data from a sample of middle-aged to older individuals at elevated risk for AD, assessed its longitudinal validity in serial PET scans, and examined its predictive value for forecasting longitudinal cognitive decline. The estimated amyloid staging model suggested a regional hierarchy where amyloid deposition begins in anterior and posterior cortical midline structures, lateral temporo-parietal association areas, and the inferior temporal lobe (stage I), and then sequentially affects the remaining association cortex, the striatum, primary sensory-motor areas, and finally the medial temporal lobe and thalamus (Fig. 1). Individual stage transitions in longitudinal PET data largely adhered to this cross-sectionally estimated staging model (Fig. 2), and complementary analysis of longitudinal amyloid accumulation in individuals without any evidence of regional amyloid deposition at baseline corroborated an early affection of the estimated stage I regions, particularly of the inferior temporal lobe and the posterior cingulate. An early increase of amyloid signal in temporal lobe regions has been reported in several previous studies using ^18^F-based radiotracers [37–39] and was also a consistent feature in our previously developed regional amyloid staging models based on ^18^F-florbetapir PET data from the ADNI cohort [10, 17]. While this is also consistent with early neuropathological estimates of regional amyloid progression [9], other amyloid-PET studies have more consistently pointed to the anterior and posterior cingulate as the earliest amyloid accumulating regions in AD [8, 40, 41]. Regarding this discrepancy, it was hypothesized that the early amyloid-PET signal increases in the temporal lobe may reflect some sort of “physiological” age-related amyloid deposition, whereas amyloid accumulation in anterior and posterior midline structures is more closely associated with progressive AD pathology [17, 39].

The overall progression pattern from cortical association areas over primary sensory-motor areas to the medial temporal lobe and subcortical structures is largely consistent with our previously estimated staging models for ^18^F-florbetapir PET. One notable difference of the current results is the relatively early affection of the striatum (stage II), preceding affection of primary sensory-motor areas and the medial temporal lobe (stage III). Early amyloid deposition in the striatum has been reported in PiB-PET data from autosomal-dominant variants of AD [42–44]. In contrast, striatal amyloid deposits are estimated to correspond to relatively advanced stages of amyloid pathology in sporadic AD (Thal phase 3) [4]. One previous PiB-PET staging study found elevated striatal PiB-PET signal only among individuals who already had elevated signal in neocortical association areas, but primary sensory-motor cortical areas or the medial temporal lobe were not assessed in that study [45]. It remains to be determined whether the observed difference in striatal involvement relates to actual differences in regional radiotracer binding characteristics or rather reflects specifics of the different cohorts used for estimating the staging models.

Similar to our previous staging studies [10, 11, 17], the current PiB-PET findings suggest that the estimated amyloid staging model provides a higher sensitivity for early amyloid detection compared to a more conventional binary classification of subjects into amyloid-negative or amyloid-positive categories. Only 37.5% of the stage I participants were categorized as amyloid-positive by a standard global amyloid threshold proposed for binarization of the ^11^C-PiB-PET DVR data [8].

A major strength of our current study is that we were able to study the effect of the estimated amyloid stages on long-term cognitive trajectories assessed over an average of 6.5 years of clinical follow-up. Here, we could demonstrate that risk of cognitive decline was proportional to the estimated amyloid stage. While individuals with advanced amyloid stages III and IV at baseline showed a faster decline in both the PACC3 global composite and domain-specific scores, even the earlier stage II showed a significantly faster decline in executive function compared to individuals without evidence of regional amyloid pathology (stage 0). The somewhat counterintuitive finding that stage IV participants did not also demonstrate a significantly faster decline in executive function may possibly be due to the relatively low numbers of participants categorized into this stage and the high variability in domain-specific cognitive decline in the preclinical phase of AD [46]. In contrast to our current findings, some previous studies reported a relatively memory-specific cognitive decline in function of higher global amyloid-PET levels among middle-aged and older adults [47]. However, a meta-analysis across 5000 cognitively normal older individuals did not find evidence for a specific effect of amyloid accumulation on memory decline compared to other cognitive domains [48]. Interestingly, participants in stages 0 and II demonstrated a slight improvement in immediate learning and delayed recall over time (Fig. 4, Table 2), which likely reflects practice effects due to repeated exposure to the tests. Such effects have been described in cognitively normal at-risk cohorts before [49], and they are also consistent with previous findings in the WRAP cohort [50]. To compare the predictive value of regional amyloid stages with global PiB-PET signal, we conducted supplementary regression analyses replacing the regional amyloid stages with subgroupings based on a stratification of the range of standard (i.e., non-PVE-corrected) global average DVR values into five equal parts (global DVR groups 0–IV; see Supplementary table 3). Not surprisingly, the global DVR grouping was generally highly correlated with the amyloid stages in the regional staging model (Spearman’s rho = 0.78): participants who were assigned into higher DVR groups also tended to be in more advanced regional amyloid stages. However, classification based on global DVR also showed some discrepancies with regional amyloid stages, for example, global DVR group I included participants from all regional amyloid stages.

This underscores the differences between measures of global amyloid load and staging of regional amyloid accumulation. The global DVR stratification was also a significant predictor of longitudinal cognitive decline (Supplementary table 4). Regression analysis with longitudinal cognitive measures suggested that global DVR group III showed a significantly faster decline on all cognitive composite scores. Global DVR group IV showed a significant decline only on delayed recall and immediate learning composite scores, although this observation is limited by the very small number of participants classified into this very advanced global amyloid stage in our study sample.

The faster decline of the global cognitive measure in stages III and IV and a more domain-selective decline in stage II are generally consistent with previous research linking region-specific amyloid deposition to longitudinal cognitive decline in non-demented individuals, although previous studies typically examined considerably older individuals and over shorter follow-up intervals. In our recent ^18^F-florbetapir PET-based amyloid staging study, we found that higher amyloid stages (from stage II onwards) were associated with a higher risk for progression to mild cognitive impairment in cognitively normal older individuals and subjective memory complainers from two different cohorts [13]. A voxel-based imaging study found that the earliest amyloid-related episodic memory decline among cognitively normal individuals associated with spatially circumscribed increases in regional amyloid-PET signal in the medial and lateral parietal neocortex [51]. Other studies have linked amyloid spread to the striatum and other subcortical regions with a higher risk of cognitive decline among non-demented individuals [45, 52]. Thus, when subjects were stratified into three stages according to their neocortical and striatal/subcortical radiotracer uptake values, individuals in the most advanced stage with high neocortical and high subcortical signal demonstrated a significantly faster longitudinal decline in cognitive performance compared to both individuals without evidence of amyloid deposition and those with only neocortical amyloid deposition [45, 52]. Here, we extend these findings to a more comprehensive data-driven regional amyloid staging scheme and a considerably younger at-risk population, thus emphasizing the potential clinical relevance of early detection of regional amyloid deposition even among healthy middle-aged individuals at risk for AD.

### Strengths and limitations

A principal limitation of our study is that although the initial sample size was relatively large, the final number of participants categorized into the different amyloid stages was relatively low, which was also reflected in a low proportion of globally amyloid-positive individuals in the cohort (15%). Previous studies on the WRAP cohort have reported relatively higher rates of amyloid positivity (approximately 20% depending on the assessed subcohort and study visit), which could be due to the use of different cortical masks for calculating the global average signal [53, 54]. The regional staging approach employed in our study uses several methodological settings that aim to increase the correspondence of regional PiB-PET measurements with actual amyloid accumulation, including the use of DVR images [55], PVE correction [28, 56], and region-specific amyloid positivity thresholds [17]. In complementary analyses, we assessed the potential contributions of these methodological aspects by estimating additional amyloid staging models using alternative PET processing methods and threshold definitions (Supplementary figures 1-3; Supplementary tables 1, 5, and 6). Generally, the staging models estimated using SUVR images and non-PVE-corrected data showed only relatively minor differences compared to the main model with PVE-corrected DVR data (Supplementary figure 3), such that the respective regional amyloid positivity frequencies were highly correlated (rho ~ 0.86; Supplementary table 5). By contrast, the use of a constant universal cutoff for all brain regions had a major influence on the regional staging model, yielding low-rank correlations with the regional amyloid positivity frequencies of all other models (rho ≤ 0.22). While the highest frequencies among cortical regions were similarly observed in anterior and posterior midline regions, regional frequencies were also very high in subcortical structures, including the striatum and thalamus (54% and 95%, respectively), when using a constant cutoff. These high frequencies of amyloid positivity could likely be attributed to the high influence of universal, non-specific white matter binding in these subcortical regions embedded within the brain’s white matter. All methods used for estimating region-specific cutoffs (2D-GMM, 1D-GMM, young subsample; see Supplementary figure 1) suggested considerably higher cutoffs for these structures compared to the thresholds estimated for most cortical regions. In terms of individual staging results, all models based on alternative PET processing strategies resulted in one or two additional participants being classified as non-stageable, indicating a minor advantage of the main model (using PVE-corrected DVR maps and region-specific thresholds) in this regard.

Our employed regional amyloid staging approach also has some limitations. First, the staging model in the present study was estimated using data from a single radiotracer, which could limit its applicability to data obtained with other tracers as compared to staging models estimated across multi-tracer amyloid-PET data [57]. Second, the current staging approach assumes a single trajectory for regional amyloid accumulation across all individuals. Although the very low number of non-stageable participants argues against major deviations of regional amyloid deposition across the four larger anatomical divisions estimated by the staging model, individual variance may still be present at a more granular spatial resolution not considered by the model (e.g., variable involvement of inferior and lateral temporal, anterior medial, and posterior medial cortical regions, all represented within the same stage I in the model). Moreover, other pathologies that often co-occur with AD-typical amyloid accumulation, such as cerebral amyloid angiopathy (CAA), may contribute to individual deviations from the estimated staging model. In this context, it is interesting to note that three out of the four non-stageable participants demonstrated amyloid positivity in the occipital pole, which showed an overall low frequency of amyloid positivity in this sample but had previously been associated with CAA [58].

## Conclusion

In summary, in the current study, we applied the in vivo amyloid staging approach that was developed and replicated previously using ^18^F-florbetapir PET data to ^11^C-PiB-PET data from a relatively young cohort enriched with risk for AD. The cross-sectionally estimated staging model utilizing region-specific thresholds was largely consistent with previous models established based on ^18^F-florbetapir PET and was further supported by model-conform longitudinal stage transitions as well as by the pattern of longitudinal emergence of regional amyloid positivity in participants who had no amyloid-positive regions at baseline. Using exceptionally long clinical follow-up data we could demonstrate amyloid stage-proportional risks of cognitive decline even in this comparably young at-risk cohort. Together, these data support the robustness and clinical utility of in vivo amyloid staging for risk stratification of the preclinical phase of AD.

## Supplementary Information

**Additional file 1: **Effect of different methods for estimating regional amyloid positivity thresholds on staging models of regional amyloid progression. **Figure S1.** Regional amyloid-positivity frequencies across three methods used for estimating amyloid accumulation thresholds. **Table S1.** Correlations between amyloid-positivity frequencies based on different amyloid-positivity thresholds. **Table 2.** Sample characteristics by longitudinal amyloid progression status. **Table 3**. Correspondence between regional amyloid staging results and subgroups stratified by global DVR signal. **Table 4.** Mixed-effects regression models of longitudinal change in composite cognitive scores across groups of participants stratified by global non-PVE-corrected ^11^C-PiB-PET DVR signal. Effect of different PET processing methods on estimated staging models of regional amyloid progression **Figure S2**. Correspondence between global DVR signal extracted from non-PVE-corrected and PVE-corrected ^11^C-PiB-PET data. **Figure S3.** Regional amyloid-positivity frequencies across different PET processing methods. **Table S5.** Correlations between amyloid-positivity frequencies based on different PET processing methods. **Table S6.** Summary of individual staging results across models with different PET processing methods.

## Data Availability

The data analyzed in this study were acquired from the Wisconsin Registry for Alzheimer’s Prevention (WRAP) study. Data are available for authorized researchers upon request subject to a decision by the WRAP Science Executive Committee. For data requests, please refer to https://wrap.wisc.edu/data-requests/.

## Abbreviations

AD: Alzheimer’s disease
APOE: Apolipoprotein E
Aβ: Beta-amyloid
DVR: Distribution volume ratio
GMM: Gaussian mixture model
MCI: Mild cognitive impairment
MMSE: Mini-Mental State Examination
MRI: Magnetic resonance imaging
PACC3: Three test version of the preclinical Alzheimer’s cognitive composite
PET: Positron emission tomography
^11^C-PiB: ^11^C Pittsburgh Compound B
THEO-DEL-REC: Delayed recall composite score
THEO-EXEC-FN: Executive function composite score
THEO-IMM-LRN: Immediate learning composite score
WRAP: Wisconsin Registry for Alzheimer’s Prevention
