# Supplementary material for: In vivo staging of regional amyloid progression in healthy middle-aged to older people at risk of Alzheimer’s disease
Source: Alzheimers Res Ther. 2021 Oct 21;13:178. doi: 10.1186/s13195-021-00918-0 (PMC8532333; doi:10.1186/s13195-021-00918-0)
Supplement: Supplementary file 1 — Additional file 1: Effect of different methods for estimating regional amyloid positivity thresholds on staging models of regional amyloid progression. Figure S1. Regional amyloid-positivity frequencies across three methods used for estimating amyloid accumulation thresholds. Table S1. Correlations between amyloid-positivity frequencies based on different amyloid-positivity thresholds. Table 2. Sample characteristics by longitudinal amyloid progression status. Table 3. Correspondence between regional amyloid staging results and subgroups stratified by global DVR signal. Table 4. Mixed-effects regression models of longitudinal change in composite cognitive scores across groups of participants stratified by global non-PVE-corrected 11C-PiB-PET DVR signal. Effect of different PET processing methods on estimated staging models of regional amyloid progression Figure S2. Correspondence between global DVR signal extracted from non-PVE-corrected and PVE-corrected 11C-PiB-PET data. Figure S3. Regional amyloid-positivity frequencies across different PET processing methods. Table S5. Correlations between amyloid-positivity frequencies based on different PET processing methods. Table S6. Summary of individual staging results across models with different PET processing methods. [file 13195_2021_918_MOESM1_ESM.pdf]

Supplementary information.

In-vivo staging of regional amyloid progression in healthy middle aged to older people at risk of Alzheimer's disease

Fedor Levin[1], Irina Jelistratova[1], Tobey J. Betthauser[2,3], Ozioma Okonkwo[2,3], Sterling C. Johnson[2,3,4,5], Stefan J. Teipel[1,6], Michel J. Grothe[1,7*]

[1]German Center for Neurodegenerative Diseases (DZNE), Rostock/Greifswald, Rostock, Germany

[2]Division of Geriatrics and Gerontology, Department of Medicine, University of Wisconsin–Madison School of Medicine and Public Health, Madison, WI, USA.

[3]Wisconsin Alzheimer's Disease Research Center, University of Wisconsin–Madison School of Medicine and Public Health, Madison, WI, USA

[4]Wisconsin Alzheimer's Institute, University of Wisconsin–Madison School of Medicine and Public Health, Madison, WI, USA

[5]Geriatric Research Education and Clinical Center, William S. Middleton Memorial Veterans Hospital, Madison, WI, USA

[6]Department of Psychosomatic Medicine, University of Rostock, Rostock, Germany

[7]Unidad de Trastornos del Movimiento, Servicio de Neurología y Neurofisiología Clínica, Instituto de Biomedicina de Sevilla, Hospital Universitario Virgen del Rocío/CSIC/Universidad de Sevilla, Seville, Spain.

*Corresponding Author:

Michel J. Grothe

Unidad de Trastornos del Movimiento, Instituto de Biomedicina de Sevilla (IBiS), Campus Hospital Universitario Virgen del Rocío, Avda. Manuel Siurot, s/n, 41013 Sevilla, Spain

Email: mgrothe@us.es; Phone: +34 955 923 000

Effect of different methods for estimating regional amyloid positivity thresholds on staging models of regional amyloid progression

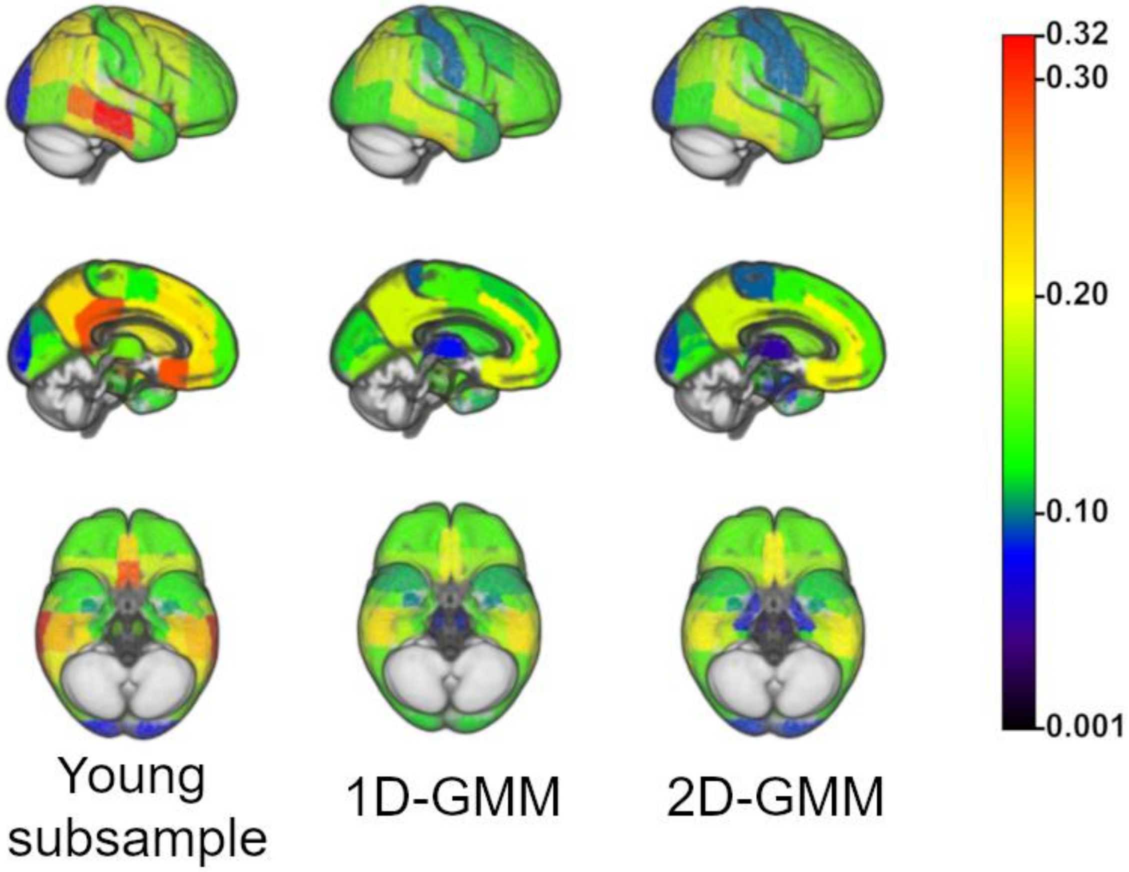

Supplementary figure 1. Regional amyloid-positivity frequencies across three different methods used for estimating regional amyloid accumulation thresholds.

Young subsample = approach estimating thresholds based on the data from a subsample of the 20 youngest APOE-ε4 negative participants without parental history of AD; 1D-GMM = one-dimensional Gaussian Mixture Model approach using regional amyloid signal only; 2D-GMM = two-dimensional Gaussian Mixture Model approach using regional and global amyloid signal.

Supplementary table 1.

Correlations between amyloid-positivity frequencies based on different amyloid-positivity thresholds.

	1D-GMM	Young subsample	Previous ^{18}F-Florbetapir based model
2D-GMM	0.779***	0.633***	0.535***
1D-GMM	-	0.574***	0.367**
Young subsample	-	-	0.19*

* p < .05, ** p < .01, *** p < .001.

1D-GMM = one-dimensional Gaussian Mixture Model approach; 2D-GMM = two-dimensional Gaussian Mixture Model approach; Young subsample = resampled frequencies using thresholds based on data from 20 youngest participants; Previous 18F-Florbetapir based model = frequencies of regional amyloid-positivity in the ADNI cohort as estimated in the previous study by Grothe et al. (1).

Supplementary table 2.

Sample characteristics by longitudinal amyloid progression status.

	Progressing	Not progressing
n	24	127
Age, years (SD)	63.8 (5.8)	60.3 (5.9)
Sex, % female	63%	69%
Education, years (SD)	17.1 (2.6)	16.7 (2.7)
APOE ε4 (%)	48%	30%
Parental history of AD (%)	63%	72%
Mean global ^{11}C-PiB DVR	1.11	1.00
Global ^{11}C-PiB DVR > 1.08	11 (46%)	9 (7%)
MMSE	29.2 (1)	29.3 (1.1)
Delayed recall composite score	0.07 (0.66)	0.13 (0.74)
Executive function composite score	-0.11 (0.76)	-0.02 (0.71)
Immediate learning composite score	0.07 (0.71)	0.08 (0.77)
PACC3 composite score	-0.07 (0.66)	0.04 (0.75)

Values for years of age, education, MMSE and cognitive composite scores are presented as means with standard deviation in parentheses. Please note that individuals with missing values were excluded for this summary.

Supplementary table 3.

Correspondence between regional amyloid staging results and subgroups stratified by global DVR signal.

	Global average DVR groups					
	Group 0	Group I	Group II	Group III	Group IV	Sum
Stage 0	167	16				183
Stage I	2	6				8
Stage II		5	2			7
Stage III		4	2	4		10
Stage IV		1	2	3	2	8
Non-stageable	1	3				4
Sum	170	35	6	7	2	

Groups 0-IV represent five groupings of participants based on the five-part division of the range of global non-PVE-corrected ^{11}C-PiB-PET DVR signal. Global DVR groupings highly correlated with the amyloid stages (Spearman's rho = 0.782, $p < 0.001$).

Supplementary table 4.

Mixed-effects regression models of longitudinal change in composite cognitive scores across groups of participants stratified by global non-PVE-corrected ^{11}C-PiB-PET DVR signal.

	Delayed recall composite score		Executive function composite score		Immediate learning composite score		PACC3 composite score	
	Estimate	t-statistic	Estimate	t-statistic	Estimate	t-statistic	Estimate	t-statistic
Intercept	-0.162	-0.25	2.652***	4.088	-0.033	-0.049	0.529	0.833
Age	-0.024**	-2.966	-0.058***	-7.082	-0.03***	-3.509	-0.041***	-5.127
Gender	0.455***	4.704	0.091	0.926	0.547***	5.393	0.512***	5.384
Education	0.057**	3.327	0.043*	2.522	0.06***	3.357	0.068***	4.047
Follow-up time, years	0.026***	3.666	-0.017**	-2.877	0.027***	3.899	-0.001	-0.136
Group I	0.094	0.661	-0.153	-1.113	-0.018	-0.123	0.007	0.049
Group II	-0.404	-1.194	-0.07	-0.217	-0.223	-0.641	-0.184	-0.578
Group III	0.018	0.063	-0.11	-0.377	0.11	0.374	-0.182	-0.673
Group IV	0.879	1.186	-0.364	-0.517	0.666	0.874	0.254	0.363
Follow-up time × Group I	-0.022	-1.289	-0.036*	-2.53	-0.007	-0.431	-0.013	-0.894
Follow-up time × Group II	0.035	0.762	-0.028	-0.752	-0.008	-0.175	-0.053	-1.384
Follow-up time × Group III	-0.209***	-5.065	-0.082*	-2.452	-0.179***	-4.455	-0.122***	-3.534
Follow-up time × Group IV	-0.231*	-2.42	0.019	0.245	-0.242**	-2.597	-0.14	-1.765

Unstandardized estimates are presented with t-statistics. * $p < .05$, ** $p < .01$, *** $p < .001$. Groups 0-IV represent five groupings of participants based on the five-part division of the range of global non-PVE-corrected ^{11}C-PiB-PET DVR signal. For interactions between the follow-up time in years and stage, Group 0 acts as a reference. Random intercepts for participants are included to account for multiple measurements.

Effect of different PET processing methods on estimated staging models of regional amyloid progression

Methods

We evaluated the impact of several PET processing methods and threshold definitions on the derived staging model. For assessing the impact of the use of region-specific amyloid-positivity thresholds on the current staging model, we estimated a comparison model in which a uniform threshold was applied to every region. The amyloid-positivity threshold was based on the previously established threshold of 1.08 (2) which was converted for use with PVE-corrected data via a regression model with the resulting value of DVR = 1.03 (Supplementary figure 2). Next, we assessed the potential effect of applying PVE-correction by deriving a staging model using non-PVE corrected PET data. Finally, we also evaluated potential differences between the main staging model derived based on ^{11}C-PiB-PET DVR data and a model based on SUVR data. Briefly, individual SUVR maps were obtained using dynamic PET frames from a 50 to 70 min window and using a cerebellar gray matter reference region. These SUVR maps were then processed identically to the DVR maps, including correction for partial volume effects using the 3-compartment "Müller-Gärtner" method in subject's native space (3, 4), and spatial normalization to the reference template space using transformation parameters from the corresponding MRI. Analogously to the model based on DVR maps, region-specific amyloid-positivity thresholds for the SUVR data were obtained using regional and global composite amyloid signal in 2D-GMM. A frequency-based staging model was then estimated and used for staging individual ^{11}C-PiB-PET SUVR profiles. The correspondence between regional amyloid-positivity frequencies of these models was assessed using Spearman rank correlations.

Results

Brain renderings illustrating the regional amyloid-positivity frequencies of the different models are shown in Supplementary figure 3, and the corresponding Spearman rank correlations for pair-wise comparisons of the models are listed in Supplementary table 5. Generally, the staging models estimated using SUVR images and non-PVE-corrected data showed only relatively minor differences compared to the main model with PVE-corrected DVR data, such that the respective

regional amyloid-positivity frequencies were highly correlated (rho ~ 0.86; Supplementary table 5). By contrast, the use of a constant universal cut-off for all brain regions had a major influence on the regional staging model, yielding low rank correlations with the regional amyloid-positivity frequencies of all other models (rho $\leq$ 0.22). While highest frequencies among cortical regions were similarly observed in anterior and posterior midline regions, regional frequencies were also very high in subcortical structures, including the striatum and thalamus (54% and 95% respectively), when using a constant cut-off. In terms of individual staging results, all models based on alternative PET processing strategies resulted in one or two additional participants being classified as non-stageable, indicating a minor advantage of the main model (using PVE-corrected DVR maps and region-specific thresholds) in this regard (see Supplementary table 6).

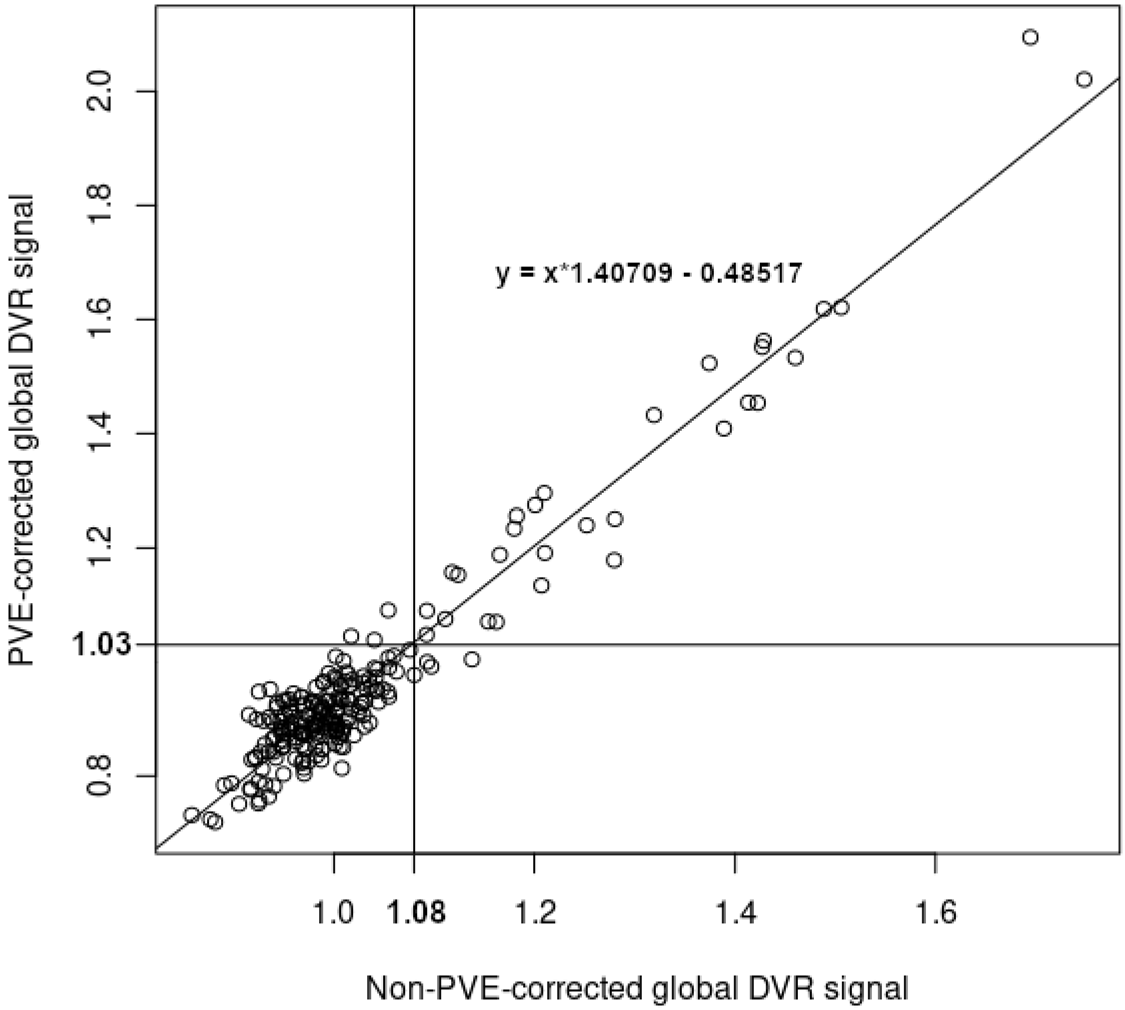

Supplementary figure 2. Correspondence between global DVR signal extracted from non-PVE-corrected and PVE-corrected ^{11}C-PiB-PET data.

A previously established threshold for amyloid-positivity of DVR = 1.08 (2) was converted to DVR = 1.03 using the linear regression equation.

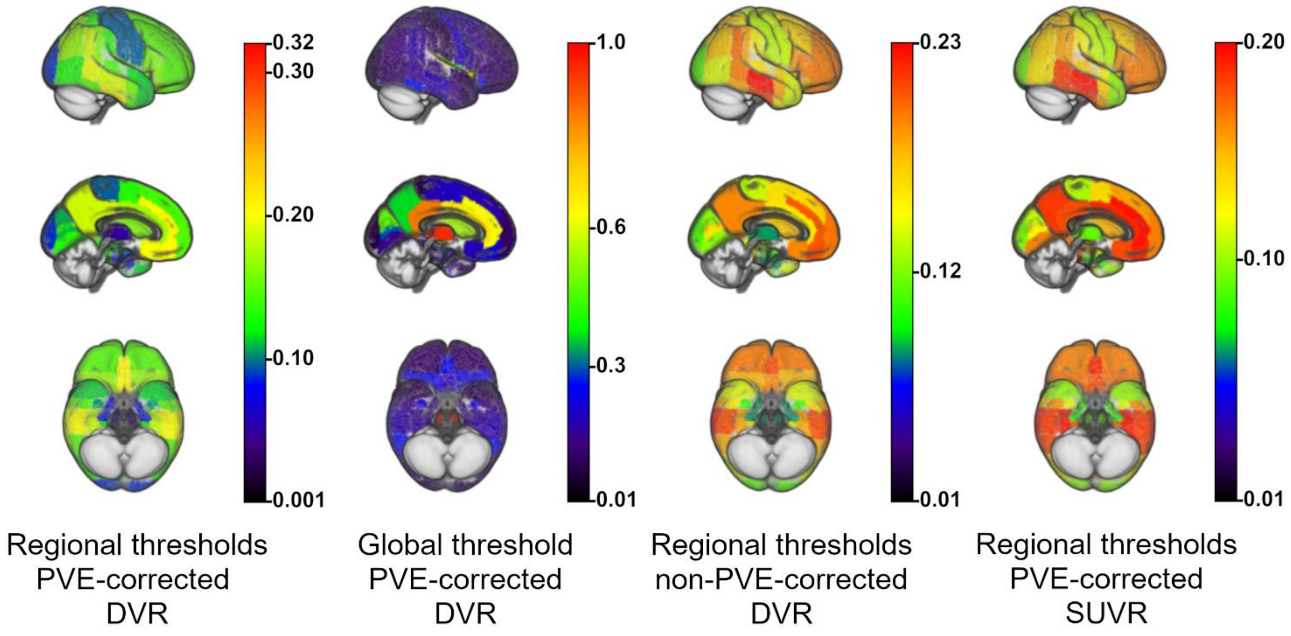

Supplementary figure 3. Regional amyloid-positivity frequencies across different PET processing methods.

Regional thresholds PVE-corrected DVR = main staging model using region-specific amyloid-positivity thresholds with PVE-corrected DVR maps, included for reference.
Global threshold PVE-corrected DVR = comparison staging model using a uniform threshold with PVE-corrected DVR maps.
Regional thresholds non-PVE-corrected DVR = comparison staging model using region-specific amyloid-positivity thresholds with non-PVE-corrected DVR maps.
Regional thresholds PVE-corrected SUVR = comparison staging model using region-specific amyloid-positivity thresholds with PVE-corrected SUVR maps.

Supplementary table 5.

Correlations between amyloid-positivity frequencies based on different PET processing methods.

	Global threshold PVE-corrected DVR	Regional thresholds non-PVE-corrected DVR	Regional thresholds PVE-corrected SUVR
Regional thresholds PVE-corrected DVR	0.224	0.862***	0.859***
Global threshold PVE-corrected DVR		0.042	0.206
Regional thresholds non-PVE-corrected DVR			0.716***

* p < .05, ** p < .01, *** p < .001.

Regional thresholds PVE-corrected DVR = main staging model using region-specific amyloid-positivity thresholds with PVE-corrected DVR maps.
Global threshold PVE-corrected DVR = comparison staging model using a uniform threshold with PVE-corrected DVR maps.
Regional thresholds non-PVE-corrected DVR = comparison staging model using region-specific amyloid-positivity thresholds with non-PVE-corrected DVR maps.
Regional thresholds PVE-corrected SUVR = comparison staging model using region-specific amyloid-positivity thresholds with PVE-corrected SUVR maps.

Supplementary table 6.

Summary of individual staging results across models with different PET processing methods

	Stage 0	Stage I	Stage II	Stage III	Stage IV	Non-stageable
Regional thresholds PVE-corrected DVR (main model)	183	8	7	10	8	4
Global threshold PVE-corrected DVR	10	47	79	52	26	6
Regional thresholds non-PVE-corrected DVR	176	8	6	9	15	6
Regional thresholds PVE-corrected SUVR	183	5	6	6	15	5

Regional thresholds PVE-corrected DVR = main staging model using region-specific amyloid-positivity thresholds with PVE-corrected DVR maps, included for reference.
Global threshold PVE-corrected DVR = comparison staging model using a uniform threshold with PVE-corrected DVR maps.
Regional thresholds non-PVE-corrected DVR = comparison staging model using region-specific amyloid-positivity thresholds with non-PVE-corrected DVR maps.
Regional thresholds PVE-corrected SUVR = comparison staging model using region-specific amyloid-positivity thresholds with PVE-corrected SUVR maps.

References:

1. Grothe MJ, Barthel H, Sepulcre J, Dyrba M, Sabri O, Teipel SJ, et al. In vivo staging of regional amyloid deposition. Neurology. 2017;89(20):2031-8.

2. Villeneuve S, Rabinovici GD, Cohn-Sheehy BI, Madison C, Ayakta N, Ghosh PM, et al. Existing Pittsburgh Compound-B positron emission tomography thresholds are too high: statistical and pathological evaluation. Brain. 2015;138(Pt 7):2020-33.

3. Gonzalez-Escamilla G, Lange C, Teipel S, Buchert R, Grothe MJ, Alzheimer's Disease Neuroimaging I. PETPVE12: an SPM toolbox for Partial Volume Effects correction in brain PET - Application to amyloid imaging with AV45-PET. Neuroimage. 2017;147:669-77.

4. Muller-Gartner HW, Links JM, Prince JL, Bryan RN, McVeigh E, Leal JP, et al. Measurement of radiotracer concentration in brain gray matter using positron emission tomography: MRI-based correction for partial volume effects. J Cereb Blood Flow Metab. 1992;12(4):571-83.
